# Expanding Cancer Prevention: Strategies Integrated into Occupational Health Surveillance

**DOI:** 10.3390/cancers17213535

**Published:** 2025-10-31

**Authors:** Giulia Collatuzzo, Alessandro Godono, Giulia Fiorini, Daniel Vencovsky, Stefano Giordani, Valentina Biagioli, Felipe Augusto Pinto-Vidal, Monireh Sadat Seyyedsalehi, Magdalena Kostrzewa, Angel Honrado, Daniele Bruno, Adonina Tardon, Dana Mates, Anna Schneider-Kamp, Eleonora Fabianova, Paolo Boffetta

**Affiliations:** 1 Department of Biomedical and Clinical Sciences (DIBIC), Università degli Studi di Milano, 20122 Milano, Italy; 2 Department of Public Health and Pediatrics, University of Torino, 10126 Turin, Italy; 3 Department of Medical and Surgical Sciences, IRCCS S. Orsola University of Bologna, 40138 Bologna, Italy; 4 RPA Europe Prague s.r.o., 130 00 Prague, Czech Republic; 5 Department of Medical and Surgical Sciences, University of Bologna, 40124 Bologna, Italy; 6 Past—Oncologia Territoriale—AUSL, 40124 Bologna, Italy; 7 Associazione Onconauti Bologna, 40133 Bologna, Italy; 8 WeDo | Project Intelligence Made Easy, S.L., 08018 Barcelona, Spain; 9 Health Research Institute of Asturias (ISPA), 33011 Oviedo, Spain; 10 Faculty of Medicine, Universidad Nebrija, 28040 Madrid, Spain; 11 National Institute of Public Health, 050463 Bucharest, Romania; 12 Department of Business and Management, University of Southern Denmark, DK-5230 Odense, Denmark; 13 Occupational Health Department, Regional Authority of Public Health, 497556 Banská Bystrica, Slovakia; 14 Stony Brook Cancer Center, Stony Brook University, Stony Brook, NY 11794, USA; 15 Department of Family, Population and Preventive Medicine, Renaissance School of Medicine, Stony Brook University, Stony Brook, NY 11794, USA

**Keywords:** cancer prevention, cancer screening, health promotion, occupational health surveillance, workplace, total worker health

## Abstract

**Simple Summary:**

In a landscape where population aging and scarce control of modifiable risk factors contribute to unfavorable cancer patterns, strategies aimed at raising awareness and increasing participation in cancer screening are urgent. Several barriers related to both individual and environmental factors impair the participation in available cancer prevention screening. This critical issue particularly concerns disadvantaged subgroups. We offer a novel viewpoint of extended occupational-based cancer prevention, which therefore targets workers and directly involves occupational health providers. The structured and longitudinal schedule of occupational health surveillance offers a unique setting for the implementation of cancer prevention programs. Such an approach would contribute to the overall health and wellbeing of the workers and might contribute significantly to cancer control at the public level.

**Abstract:**

Participation in cancer prevention programs is suboptimal. Socioeconomic backgrounds play a role in cancer awareness and prevention programs. We conducted a narrative review, summarizing the evidence on the integration of cancer prevention extended to non-occupational risk factors at the workplace. Cancer prevention programs include screenings (colonoscopy, mammography, Pap-test), vaccinations (anti-HPV, anti-HBV), and interventions focused on lifestyle changes. Such strategies may face several barriers related to individual or environmental factors. The workplace is potentially an ideal setting for implementing extended cancer prevention strategies because (i) occupational health surveillance (OHS) targets adults, including hard-to-reach subgroups; (ii) it is structured, with health records and exams for risk assessment; (iii) it offers a key chance to promote cancer awareness and prevention through direct worker–physician interaction. Such an innovative approach requires a coordinated effort to build professional networks and manage high-risk workers. Its successful implementation depends on financial support and the active involvement of physicians, employers, and workers. Occupational-based cancer prevention represents a novel and promising strategy, though its feasibility and cost-effectiveness need to be assessed through large-scale studies.

## 1. Introduction

The growing focus on prevention in the medical field has contributed to reducing morbidity and mortality from major diseases, including cancer [1]. An essential component of effective cancer prevention programs is the identification of modifiable risk factors [2]. While cancer etiology has traditionally been attributed to genetics and chance, most cancers are due to modifiable risk factors, including lifestyle, nutritional, infectious, occupational, and environmental exposures [3,4,5], implying that many cancers are avoidable [4].

Despite the implementation of some preventive strategies, the proportion of avoided cancer is still unsatisfactory due to several challenges [4,6,7,8,9,10]. These include disparities in cancer prevention program availability, suboptimal adherence to cancer screening and prevention measures, resistance to lifestyle changes, low health literacy, barriers to healthcare access, costs of prevention initiatives, and the slow integration of scientific evidence into medical practice [9]. Cancer prevention programs must address these challenges by offering accessible, cost-effective, noninvasive, and easy-to-use services, favoring population participation [10,11].

Tailoring programs based on risks enhances cost-effectiveness and allows for more targeted efforts [12] in specific settings [13,14] and specific demographic groups [15,16,17,18,19].

The workplace is a promising setting for cancer prevention initiatives [20,21,22,23], which could be part of structured occupational health surveillance (OHS) [24]. OHS often focuses narrowly on occupational hazards [24]. Prevention or minimization of exposure to carcinogens at the workplace is a major required measure, as per current European legislation [25,26]. Besides this, many employers voluntarily expand their occupational health services to include broader preventive measures.

The “Total Worker Health” (TWH) approach, developed by the National Institute for Occupational Safety and Health (NIOSH) [27], emphasizes protecting workers not only from occupational risks, but also from non-occupational risk factors of chronic diseases that impact their overall wellbeing. The integration of wide cancer prevention strategies at the occupational level aligns with the TWH approach. It implies (i) interventions also targeting non-occupational cancers, and therefore (ii) extended to all workers.

Studies suggest that workplace-based interventions increase cancer screening engagement and empower workers to adopt healthier lifestyles, benefiting both individuals and their families [28]. While several studies describing workplace-based cancer screening have been published, the integration of cancer prevention interventions into OHS has not been purposed as a model to be fully and systematically implemented.

We developed the project Cancer Prevention at Work (CPW) as an interventional pilot study targeting workers from different European countries (Italy, Spain, Romania, and Slovakia) for awareness and prevention of infection-related cancers [29]. This project, funded by the European Union, has the purpose of investigating the feasibility of workplace-based cancer prevention strategies to improve equitable access to cancer prevention initiatives and promote workers’ health [29].

In this narrative expert review [30], we explore the integration of wide cancer prevention strategies into workplace settings as a novel approach, in the framework of the CPW project aims [29], which might deserve attention as a potential effective way to engage the adult population with publicly available cancer prevention initiatives.

## 2. The Role of the Occupational Physician on Population Health

The European healthcare landscape is currently facing a doctor shortage, which became evident with the COVID-19 pandemic [30]. A sizeable proportion of the European Union (EU) population does not regularly engage with their general practitioner (GP), whilst many countries require employers to ensure that workers are regularly seen by an occupational physician [31]. This implies limited opportunities for offering cancer screening and vaccination. For example, Eurostat data show that, in 2017, 24% of the EU population did not see their GP at all [32]. Recent decreases in GP consultation rates have been reported in England [33]. GP consultation rates vary between EU countries, with those characterized by the lowest primary care engagement and highest OHS frequency being best suited for extended cancer prevention. In 2019, the percentage of the population that did not see their GP for more than 12 months ranged from around 15% in France and Belgium to 49% in Romania [34]. On the other hand, in some countries, OHS is widely accessed by employees, including for non-work-related health issues [35]. Factors such as no cost for employees and service availability likely contribute to the high participation of workers in OHS [35].

These features present a valuable opportunity to expand the scope of occupational medicine to include larger preventive healthcare measures, according to a TWH model. Specifically, the occupational physician could play a significant role in primary prevention of both occupational and non-occupational cancers, complementing GP activity and contributing to public health.

## 3. Avoidable Cancers Prevention

Cancer prevention encompasses three key levels, primary, secondary, and tertiary, all designed to reduce cancer-related mortality by implementing timely and effective strategies. Table 1 [4,8,36,37,38,39,40,41,42,43,44,45,46,47] summarizes possible cancer-specific prevention strategies, which include lifestyle and behavioral interventions, health education, nutritional education and counseling, vaccinations, as well as medical examinations and/or imaging within cancer screening programs.

Occupational cancers, those cancers causally linked to professional exposure to carcinogens, are part of the fraction of cancer that would be avoidable. It is therefore highly important to include occupational history in daily clinical practice, in order to capture comprehensively the cancer risk profile of the individual.

The occupational physician, on the other hand, plays a key role in the reduction in cancer risk in workers: this figure might influence workers’ choices of healthy behaviors, including quitting smoking, reducing alcohol consumption, and engaging in regular physical activity. Also, the identification of high-risk workers (e.g., sex, age, body mass index, lifestyle habits, family history of cancer) allows the occupational physician to refer individuals to specialists for consultancy, or to suggest instrumental exams such as endoscopy [27]. Furthermore, the occupational physician can substantially contribute to the increase in cancer screening and vaccination uptake, by raising awareness and spreading health literacy among the working population.

Globally, three major cancer screening programs are widely implemented [1]: mammography (breast cancer prevention), colonoscopy (colorectal cancer prevention), and Pap-test (cervical cancer prevention). These screening initiatives form the cornerstone of secondary prevention, enabling early detection and, thereby, timely and effective treatment.

Vaccines targeting carcinogenic infections have emerged as transformative tools in primary cancer prevention. For example, the introduction of the vaccine against the hepatitis B virus (HBV) in 1982 led to a 98% reduction in infections among US healthcare workers between 1983 and 2010 [48]. In Taiwan, the risk of hepatocellular carcinoma significantly decreased among individuals born after the 1984 introduction of the anti-HBV vaccine [49]. Similarly, the vaccine against human papillomavirus (HPV) has dramatically reduced cervical cancer incidence [45]. Scaling global anti-HPV vaccine coverage to 80–100% could prevent 6.7–7.7 million cervical cancer cases over the next 50 years [45]. Despite promising well-documented outcomes, vaccination rates remain suboptimal in some countries and among disadvantaged groups. Japan’s hesitancy toward the anti-HPV vaccine [50,51], fueled by negative media coverage and cultural taboos, led to a dramatic drop in coverage to less than 1% in 2018 [50,51]. Proactive anti-HPV vaccine recommendations were reinstated in 2022, and initial vaccination rates among adolescent girls rose to 31% by 2023 [50,51]. This case underscores the critical role of public perception, communication strategies, and policy frameworks in shaping vaccine uptake and public health outcomes [50,51]. In addition, the proven causal relationship of HPV infection with cancers at other locations than the cervix, including oropharyngeal and anogenital locations, highlights the exceptional primary preventive importance of anti-HPV vaccination in both men and women [52]. To overcome persistent challenges with less than optimal coverage, tailored vaccination strategies targeting high-risk populations and marginalized groups are essential.

## 4. Social Determinants of Health and Cancer Prevention Disparities

**Figure 1:**
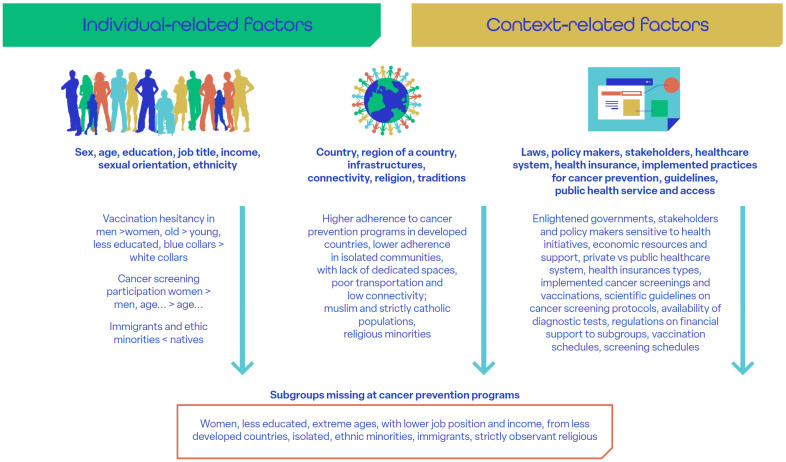
Social determinants of health [18,54,55,56,57,58,59,60,61,62,63,64,65]. (This is original material).

Recent data show increasing absolute numbers of new cases and deaths [53], mainly due to population aging, despite the ongoing downtrend of cancer incidence in most countries worldwide. Socioeconomic disparities in cancer prevention persist despite the progressive expansion of public health policies and services aimed at equity [9]. Social determinants of health include individual demographic and economic factors (e.g., sex, age, employment) [54], sociocultural context (family, support networks, education, local culture, religion) [15,18,54,55], and governmental policies (health insurance, access to screening) [56,57,58]. These determinants significantly impact adherence to implemented cancer prevention services (Figure 1 [15,18,54,55,56,57,58,59,60,61,62,63,64,65]). For instance, participation in cervical cancer screening and HPV vaccination is notably lower in low- and middle-income countries. This is, in part, due to taboos and religious constraints [56,61,62], but also to economic factors such as vaccine pricing and costs for journeys to vaccination centers [54]. At the same time, vulnerable populations, including certain groups of immigrants and other individuals of low socioeconomic status, are disproportionately affected by cancer due to increased exposure to risk factors, such as smoking, alcohol use, and poor diet [58].

Addressing cancer disparities requires culturally sensitive, evidence-based interventions [63] delivered through diverse channels [63,64,65,66], including libraries [64,65], public events, and informal community networks [66]. Such “informal” channels outside the hospital borders have the potential to improve health education and provide access to health services to a wide heterogeneous public composed of individuals with varying ages and social backgrounds, raising awareness of cancer prevention and increasing participation in cancer screening programs. In this framework, the occupational setting offers unique opportunities for cancer prevention [28,67].

The integration of extended cancer prevention into OHS could have a ripple effect on workers’ households too, spreading health education and increasing the overall participation of the general population in cancer prevention initiatives [67,68]. Moreover, occupational factors and employment status [58] are among the main social determinants of health and related disparities, requiring an aware and proactive attitude of the employers and the healthcare providers in leveraging health inequities, recognizing health as a value for business and profitability [69].

Extended cancer prevention also has the potential to address some of the imbalances in primary care utilization between men and women, as well as different age cohorts. Data from Eurostat (2019) [34] indicate that GP consultation rates at the EU level are generally higher among women than men. In 2019, the proportion of men who declared that they had not seen their GP in the preceding 12 months ranged from around 19% in Belgium to 55% in Romania, whilst the corresponding proportion of women ranged from 11% in France to 44% in Romania. This is further confirmed by Song et al. [70], who examined 2019 data on GP consultations in the UK and concluded that, whilst overall 23% of the population did not consult their GP at all in the last 12 months, the frequency of GP consultations was higher among women and those aged ≥75. Baker provides consistent data for UK people of working age (<65 years old) in 2022 [71]. However, when it comes to consultations at OHS, both men and women appear to visit with similar frequency. Therefore, a proportion of the general population might visit the occupational physician more often than their GP.

Targeting workers with extended cancer prevention programs would allow us to reach heterogeneous populations, including vulnerable subgroups who often remain uncovered by health services and reluctant subjects. This would make workplaces a cornerstone for broader healthcare initiatives [72].

## 5. The Workplace as a Setting for Cancer Prevention Programs

Occupational medicine practice is highly heterogeneous by country and implemented to different extents. Workers exposed to occupational carcinogens are subjected in many countries to OHS scheduled with specific timing and including targeted exams [24]. Employers can also offer their employees additional health check-ups through the occupational health service [72], as well as interventions aimed at health promotion. However, interventions aimed at reducing the risk of cancer due to non-occupational risk factors are not required in any country. The cognition of general health and wellbeing as a critical aspect to protect and promote among workers has become increasingly widespread [72]. This confers a central role to the occupational physician in protecting the workers from developing chronic diseases, which can compromise their work ability [27]. Such a view strengthens the role of occupational health personnel as health educators and active promoters of a healthy lifestyle [27].

The literature offers large evidence on occupational interventions which positively impacted healthy choices, based on the TWH approach [27,68,73,74,75]. With regard to cancer prevention, some examples of occupational-based interventions have been proposed, including breast, gastric, colorectal, and cervical cancer screening [28,29,67,68]. A recent systematic review has summarized the overall effectiveness of workplace-based interventions, which were found to enhance the overall knowledge and uptake of cancer screening tests [28]. Some studies have also addressed the primary prevention of cancer among workers, mostly addressing skin cancer through the use of sun protection equipment [76]. Notably, interventions that provide information (e.g., campaigns for sensitization, educational programs on cancer prevention) and instruments (e.g., vaccinations, screening tests) to the workers carry a durable benefit: they empower workers with a deeper understanding of cancer risk factors and their possible preventive measures, generating a healthier population at a lower risk of developing cancer [75].

**Figure 2:**
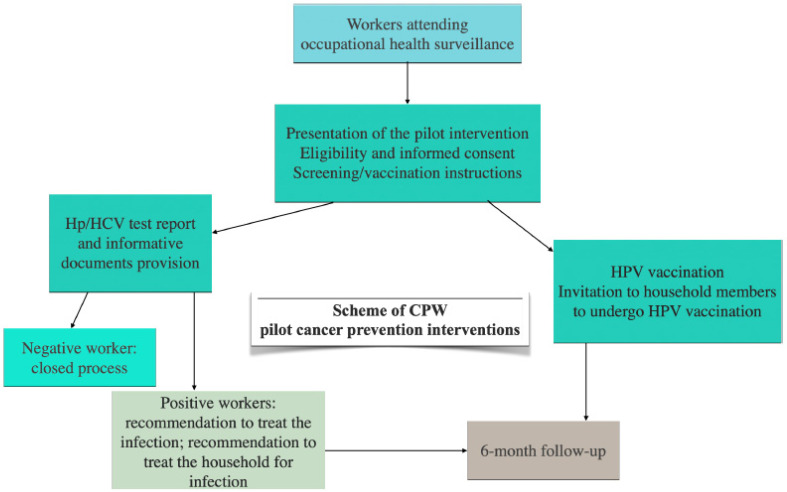
Scheme of Cancer Prevention at Work—CPW pilot interventions as an example of implementation of occupational-based cancer prevention strategies [29]. (This is original material).

While further evidence is needed for the implementation of standardized OHS-based cancer prevention programs, some protocols have been proposed [29,67,68]. In particular, the CPW project [29], as part of the implementation research projects of the EU Cancer Mission, is assessing the feasibility of cancer prevention strategies integrated into OHS, with a focus on three pilot cancer prevention interventions for infectious-related cancer control: screening of Helicobacter pylori and Hepatitis C virus (HCV) infections, and anti-human papillomavirus (HPV) vaccination. The scheme of the threepilot cancer prevention interventions is shown in Figure 2. Job areas of CPW-targeted workers include manufacturing activities, financial and insurance activities, and health and social welfare.

Such strategies, if successfully implemented, might support public health interventions already established. For example, the age range of the working population partially differs from that of currently recommended screenings (e.g., 50–75 for colorectal cancer screening). However, the progressive postponement of retirement age to over 65 years old makes occupational-based screening largely overlap, in terms of age range, with existing public health programs. In this regard, future research directions will include the assessment of potential divergences emerging at the workplace and at the population level.

Table 2 highlights the key characteristics of a screening program integrated with OHS. For each of them, the challenges, but also the opportunities, that can be obtained as a result of this particular screening setting are listed. A key aspect of occupational medicine is the regularity and structure of OHS [24,26,77,78,79,80,81], scheduled based on the occupational risk profile of each worker. OHS is mandatory and free for all workers [24,26], guaranteeing equity and reaching different strata of the general population, including minorities and less affluent subgroups. Also, OHS includes follow-up visits and longitudinal monitoring of the worker [24,26], which are both important in cancer prevention programs. Questionnaires for risk stratification and medical tests (e.g., blood and urine samples, hearing checks) are often included in OHS [24,26]. Cancer risk stratification of the worker could easily be performed through specifically designed questionnaires, while tailored tests could be administered based on risk profiles acquired during the OHS. The collection of information aimed at creating a “cloud” of personal health data is in line with a more personalized model of medicine. However, this raises concern regarding confidentiality [79]: the workers might be hesitant to share some personal information out of the fear that they could be reported to the employer [82]. A multidisciplinary team (including healthcare providers, general practitioners, nutritionists, physiotherapists, psychologists, and other experts) would be needed for successful workplace-based cancer prevention programs, where the professionals cooperate in different phases (pre-test, management of the test results, follow-up) [77]. The positive impact of interaction between different professionals has been documented, especially in the return-to-work of disabled or chronically ill subjects [77,80]. A systematic review of workplace interventions led by healthcare professionals on cardiovascular risk described higher success of interventions that were multicomponent, tailored to high-risk populations, offering motivational support and face-to-face counseling, and characterized by longitudinal follow-up [80]. The expansion of the literature regarding occupational interventions towards cancer risk could clarify the role of these characteristics in the success of workplace-based cancer prevention programs. An occupational-based cancer prevention program would require a high level of commitment, both from the employer and the medical side [78]. In the meantime, occupational health providers and employers can jointly increase participation rates in cancer prevention programs by motivating the workers to engage in health practices [69,78]. Residual disparities may also occur [58]: unemployed individuals would not benefit from these interventions; if the implementation of such programs is restricted to individual companies, some workers might remain excluded from these benefits. To avoid this problem, policy makers could propose to introduce cancer prevention as a mandatory component of OHS.

## 6. Integrating Cancer Prevention in Occupational Health Surveillance

To date, evidence on the systematic integration of cancer prevention programs within the workplace is few and far between. As a consequence, discussing its possible implementation is mainly theoretical and based on a few empirical examples.

Evidence highlighted the importance of resource availability (in terms of funds, personnel, and integration with public services) and multidisciplinary experts’ support in allowing employers to run health promotion programs in their enterprises [28,29,78]. Stakeholders of workplace-based extended cancer prevention programs might include government agencies (e.g., national and regional health ministries), health insurance systems or funders, and patient advocacy groups, rather than public health institutions or the GP; in addition, laboratories or diagnostic providers might also support such initiatives and be directly involved in the risk assessment process through the use or analyses of screening tests [29].

Next, specific professionals dedicated to the optimal implementation of such programs and workers’ engagement should be trained for this scope, likely involving multidisciplinary figures [29,83]. A successful example of a personalized approach that includes cancer prevention strategies targeting workers has been designed by the Onconauti Association, established within several centers in Italy [83]. It promotes education of all the workers around health and cancer prevention strategies and clinical management of high-risk workers through several health professional interventions, including nutritional and psychological counseling, physiotherapy, and body–mind practice sessions [84,85,86]. The work of this no-profit association is consolidated into the Italian medical practice of different provinces and collaborating healthcare centers and is financially supported by the government, with the possibility to maintain and expand its activity.

Other larger initiatives have also taken place in Europe, e.g., the European Network for Workplace Health Promotion (ENWHP) [87]. As stated through ENWHP reports, recommendations for optimal workplace health promotion should be developed within multiple levels (e.g., EU level, national and governmental level, intermediary level), identifying different actions to be undertaken by different organizations [88].

Although each country or context may differ in how cancer screening programs are organized and how occupational health is delivered, as well as in the regulatory frameworks, a shared vision and coordinated goals can enable overcoming those differences and foster effective implementation of an integrated cancer prevention and occupational medicine system [88]

Workplace-based campaigns aiming to raise health awareness are carried out at large scale. Days dedicated to work safety courses and training are already part of common workplace calendars in several countries [89,90], offering a model for cancer education interventions. However, these do not include extended cancer prevention education [89,90]. The organization of educational courses regarding overall cancer risks and prevention represents a promising avenue for raising awareness among workers [89,90,91,92].

The full and effective integration of extended cancer prevention into occupational medicine practice would require particular attention to addressing the needs of the workers at high oncological risk. In fact, a proper and adequate integrated system would cover risk profiling and clinical management of high-risk workers. To this end, access to specialized care and second-level diagnostics is a key element. Connectivity plays a crucial role [83,87,92]: ideally, private occupational medicine should be linked to a network of different professionals, including the general practitioners of the workers, through established channels. In a private healthcare system, this would require the engagement of stakeholders, with a primary role for private occupational health services in establishing agreements with specialized local organizations and health centers. In other words, occupational physicians should be enabled to provide the workers with clinical pathways tailored to specific risk profiles assessed through the OHS [83,93]. The close collaboration between local organizations and political offices, government agencies, social services, and advocacy and supporting groups in providing financial support and shaping cancer prevention policies is of utmost importance [83,87,88].

Although extended cancer prevention can entail costs for employers, it also has the potential to bring benefits. Overall, a healthy workforce offers advantages to employers, including increased worker productivity, as well as reduced absenteeism, insurance contribution, and sick leave payments [94]. Indirectly, employers at large can also benefit from a reduced demand to fund the national healthcare system via taxes due to a reduced cancer burden. Extended healthcare provision at the workplace can result in improved corporate reputation. Wider research on Corporate Social Responsibility (CSR) shows that, more generally, corporate reputation is one of the drivers of company performance and value [95,96] and can have a positive impact on staff retention and satisfaction [97].

The main benefit for employers arises from reduced incidence of cancer among workers. Recent impact assessments within the field of occupational health and safety [94] have employed the median cost to employers associated with a single case of a high-severity accident or disease, as estimated in [98], as a proxy for the costs to an employer of a worker undergoing cancer treatment. When adjusted to 2024 prices using the Harmonised Index of Consumer Prices [99], this cost is estimated to be around EUR 15,700 per worker diagnosed with cancer.

Worker absence can have significant implications for employers, as a temporary or permanent replacement for the affected worker has to be found and paid. These costs are often modeled using the concept of a ‘friction period’ that focuses on the disruption during the time period required for adjustment [100,101]. This period can be significant; for instance, a recent survey of workers diagnosed with cancer reports an average absence of 15 weeks, equating to 75 working days for a full-time employee [102].

Despite the fact that some cost savings may occur after an employee has left the company or retired, employers are still expected to benefit financially from a reduction in cancer incidence among their workforce in the long-term. Research shows that the share of workers remaining with the same employer for 10 years or more is around 50% [103]. Whilst the majority of cancer cases (65%) are diagnosed in individuals aged 65 and older, estimates from the European Cancer Information System (ECIS, 2025) [104] based on data from cancer registries and administrative sources indicate that a substantial share of cancer incidence occurs in the working-age population (34% based on ECIS data for individuals aged 20–64).

## 7. Conclusions

In a landscape where population aging and scarce control of modifiable risk factors contribute to unfavorable cancer patterns, strategies aimed at raising awareness and increasing participation in cancer screening are urgent. While the multidisciplinary and fragmented publishing record surrounding the topic make a systematic review approach difficult, the present work pools together the different expertise of the reviewers and is clinically anchored in an international multi-center project, CPW, piloting innovative integration of cancer prevention strategies into OHS in Europe.

In particular, cancer prevention programs could be integrated into regular OHS and corporate welfare, according to the TWH model. Notably, this innovative approach would benefit from multiple expertise and the involvement of different institutions and stakeholders, where the tailored clinical management of high-risk workers is the turning point to optimize the results in terms of cancer prevention.

To date, a few studies have focused on the implementation of cancer prevention interventions at the occupational level, showing promising results. The cost-effectiveness and feasibility of such innovative models need to be assessed in different sociocultural and working contexts in order to provide scientific evidence for their possible large-scale implementation. Interventional and stepped-wedge studies with longitudinal designs that provide high-quality data are required.

## Figures and Tables

**Table 1:** Examples of cancer prevention strategies by cancer types.

Cancer Type	Main Modifiable Risk Factors	Prevention Strategy			OHS Contribution	References
		Primary	Secondary	Tertiary		
**Lung cancer**	Smoking Second-hand smoke Occupational exposures	No smoking/stop smoking Avoid exposure to second-hand smoke Limit occupational exposures Use of PPE at the workplace	Low-dose CT	Rehabilitation programs Return to work programs Psychological support Nutritional counseling Promotion of physical activity and healthy lifestyle Smoking cessation Personalized support via telemedicine Sexual recovery	Occupational exposure limits PPE provision and check Quitting smoking counseling contact Risk profiling	Chow et al., 2012 [8]; Wolf et al., 2024 [36]; Loomans-Kropp and Umar, 2019 [37]; de Boer et al., 2024 [38]
**Breast** **cancer**	Obesity Low physical activity Alcohol No breastfeeding Reproductive factors	Promotion of healthy lifestyle and physical activity Health education at any age Promotion of breastfeeding	Mammography Breast ultrasound Mammotome Regular self-examination of the breast		Nutritional counseling Family history investigation Investigation and recommendation of screening and self-examination Physical activity indications Alcohol use investigation and education Recommendation of lifestyle changes Weight monitoring Risk profiling	Chow et al., 2012 [8]; Loomans-Kropp and Umar, 2019 [37]; de Boer et al., 2024 [38]; Farkas and Nattinger, 2023 [39]; Arends J et al., 2017 [40]; Campbell et al., 2019 [41]; Galiano-Castillo et al., 2016 [42]
**Colorectal cancer**	Obesity Low physical activity Diet Alcohol Smoking	Promotion of healthy lifestyle and physical activity Health education at any age Nutritional counseling Use of low-dose aspirin Microbiota analysis	Colonoscopy Rectosigmoidoscopy FOBT		Nutritional counseling Family history investigation Investigation and recommendation of screening Physical activity indications Recommendation of lifestyle changes Weight monitoring Risk profiling	Chow et al., 2012 [8]; Loomans Kropp and Umar, 2019 [37]; Arends J et al., 2017 [40]; Campbell et al., 2019 [41]; Ladabaum et al., 2020 [43]
**Gastric cancer**	Helicobacter pylori Smoking Diet Alcohol	Hp test and treatment Nutritional counseling No smoking/stop smoking	EGDS with biopsy sampling Gastric panel (pepsinogen, gastrin, and Hp IgG)		Hp test-and-treat recommendation Nutritional counseling Recommendation of lifestyle changes Weight monitoring Risk profiling	Collatuzzo and Boffetta, 2023 [4]; Chow et al., 2012 [8]; Loomans-Kropp and Umar, 2019 [37]; de Boer et al., 2024 [38]; Arends et al., 2017 [40]; Shah et al., 2025 [44]
**Cervical cancer**	HPV Smoking	HPV vaccination Sex and health education No smoking/stop smoking Regular PAP and HPV test	PPA test HPV test		Investigation and recommendation of screening HPV vaccination Recommendation of lifestyle changes Sexual health education Risk profiling	Collatuzzo and Boffetta, 2023 [4]; Chow et al., 2012 [8]; Simms et al., 2019 [45]
**Liver** **cancer**	HCV HBV Alcohol Obesity Low physical activity	HBV vaccination HCV test Promotion of healthy lifestyle and physical activity Health education at any age Serology Nutritional counseling Support for alcohol use disorder	Abdominal ultrasound CT Biomarkers (AFP)		HBV vaccination HBV and HCV screening Alcohol use investigation and education Nutritional counseling Physical activity indications Recommendation of lifestyle changes Weight monitoring Risk profiling	Collatuzzo and Boffetta, 2023 [4]; Chow et al., 2012 [8]; Loomans-Kropp and Umar, 2019 [37]; de Boer et al., 2024 [38]; Arends et al., 2017 [40]; Campbell et al., 2019 [41]; Yang et al., 2019 [46]
**Prostate cancer**	Obesity Low physical activity	Promotion of healthy lifestyle and physical activity PSA screening Clinical prostate examination	TRUS		PSA monitoring Physical activity indications Weight monitoring Recommendation of lifestyle changes	Loomans-Kropp and Umar, 2019 [37]; de Boer et al., 2024 [38]; Campbell et al., 2019 [41]; Pinsky and Parnes, 2023 [47]

OHS, Occupational Health Surveillance; CT, computed tomography; FOBT, fecal occult blood test; EGDS, esophagogastroduodenoscopy; AFP, alpha-fetoprotein; PSA, prostate-specific antigen; TRUS, transrectal ultrasound.

**Table 2:** Characteristics of the occupational setting from the perspective of an extended cancer prevention program.

Key Characteristics	Challenges	Opportunities	References
Heterogeneous categories of population (including less advantaged subgroups)	Non-compliance, missing unemployed	Equity, addressing socioeconomic barriers, age- and sex-specific interventions	WHO Regional Office for Europe, 2002 [24]; International Labour Office, 1998 [26]
General adult population (age range 18–65)	Specific age range for cancer screenings also covering ages older than 65	Primary prevention intervention in young adults, health awareness at the workplace with long-term repercussion also after employment age	WHO Regional Office for Europe, 2002 [24]; International Labour Office, 1998 [26]
Structured setting of OHS	Logistics and organization of testing, test results communication, visit duration	Regular follow-up and longitudinal monitoring of the worker’s health and adherence to cancer prevention	WHO Regional Office for Europe, 2002 [24]; International Labour Office, 1998 [26]
Available spaces for medical visits and tests	Availability and quality of instruments and infrastructures (e.g., laboratory)	Adequate environment for test/vaccination provision and storage	WHO Regional Office for Europe, 2002 [24]; International Labour Office, 1998 [26]
Planned administration of questionnaires and tests (drug test, alcohol test, blood sample, visual test, hearing check, etc.)	Required sample collection and preservation, test elimination	Adding cancer screening tests and cancer biomarkers to already planned tests	WHO Regional Office for Europe, 2002 [24]; International Labour Office, 1998 [26]
Interaction of different professionals (occupational physician, occupational nurse, employer, occupational safety representatives)	Multiple expertise required, need for enlightened figures, high responsibilities of the involved figures, increased workload of the occupational health providers	Network made of multidisciplinary team	WHO Regional Office for Europe, 2002 [24]; International Labour Office, 1998 [26]; Stratil et al., 2017 [77]
Hierarchic structure	Hesitancy and reluctance from employers and/or occupational physicians	Welfare policies of the company, enhanced wellbeing and support of workers and stakeholders, financial support	WHO Regional Office for Europe, 2002 [24]; International Labour Office, 1998 [26]; Jimenez et al., 2017 [78]
Medical records	Privacy issues, unfit for work	Personalized dataset, risk profiling, 4-P medicine, data cloud	Price and Cohen, 2019 [79]
Prevention of injuries, occupation-related diseases and workers’ health vulnerabilities	High effort requirement for extended occupational health approach	Total worker health (one health)	Schill and Chosewood, 2013 [27]; Jimenez et al., 2017 [77]; Price and Cohen, 2019 [79]
Focused on risk assessment and early diagnosis	Lack of treatment	Closer to clinics, vaccine administration, recommendation for further diagnostic tests, behavioral interventions (nutrition, physical activity, lifestyle care)	Schill and Chosewood, 2013 [27]; Loomans-Kropp and Umar, 2019 [37]; de Boer et al., 2024 [38]; Zusman et al., 2021 [80]
Overall healthy people	Management of high-risk subjects, missing subgroups of population unemployed for medical reasons	Cancer risk assessment, complementary role of occupational physician to general practitioner, support to public health practice on the general population	Schill and Chosewood, 2013 [27]; Stratil et al., 2017 [77]; Zusman et al., 2021 [80]; Persechino et al., 2017 [81]

## Data Availability

This review was based on public data.

## Abbreviations

The following abbreviations are used in this manuscript:

CPW	Cancer Prevention at Work
CSR	Corporate Social Responsibility
ECIS	European Cancer Information System
ENWHP	European Network for Workplace Health Promotion
EU	European Union
GP	General Practitioner
HBV	Hepatitis B Virus
HCV	Hepatitis C Virus
HPV	Human Papillomavirus
NIOSH	National Institute for Occupational Safety and Health
OHS	Occupational Health Surveillance
TWH	Total Worker Health
